# Intravenous transplantation of olfactory ensheathing cells reduces neuroinflammation after spinal cord injury *via* interleukin-1 receptor antagonist

**DOI:** 10.7150/thno.52197

**Published:** 2021-01-01

**Authors:** Lijian Zhang, Xiaoqing Zhuang, Päivi Kotitalo, Thomas Keller, Anna Krzyczmonik, Merja Haaparanta-Solin, Olof Solin, Sarita Forsback, Tove J. Grönroos, Chunlei Han, Francisco R. López-Picón, Hechun Xia

**Affiliations:** 1 Department of Neurosurgery, General Hospital of Ningxia Medical University, Yinchuan, Ningxia, China.; 2 Department of Neurosurgery, Affiliated Hospital of Hebei University, Baoding, Hebei, China.; 3 Department of Nuclear Medicine, General Hospital of Ningxia Medical University, Yinchuan, Ningxia, China.; 4 Preclinical Imaging Laboratory, Turku PET Centre, University of Turku, Turku, Finland.; 5 MediCity Research Laboratory, University of Turku, Turku, Finland.; 6 Radiopharmaceutical Chemistry Laboratory, Turku PET Centre, University of Turku, Turku, Finland.; 7 Department of Chemistry, University of Turku, Turku, Finland.; 8 Accelerator Laboratory, Turku PET Centre, Åbo Akademi University, Turku, Finland.; 9 Turku PET Centre, University of Turku, Turku, Finland.

**Keywords:** spinal cord injury, olfactory ensheathing cells, interleukin-1 receptor antagonist (IL-1Ra), neuroinflammation, PET imaging

## Abstract

**Rationale:** Olfactory ensheathing cell (OEC) transplantation has emerged as a promising therapy for spinal cord injury (SCI) repair. In the present study, we explored the possible mechanisms of OECs transplantation underlying neuroinflammation modulation.

**Methods:** Spinal cord inflammation after intravenous OEC transplantation was detected *in vivo* and *ex vivo* by translocator protein PET tracer [^18^F]F-DPA. To track transplanted cells, OECs were transduced with enhanced green fluorescent protein (eGFP) and HSV1-39tk using lentiviral vector and were monitored by fluorescence imaging and [^18^F]FHBG study. Protein microarray analysis and ELISA studies were employed to analyze differential proteins in the injured spinal cord after OEC transplantation. The anti-inflammation function of the upregulated protein was also proved by *in vitro* gene knocking down experiments and OECs/microglia co-culture experiment.

**Results:** The inflammation in the spinal cord was decreased after OEC intravenous transplantation. The HSV1-39tk-eGFP-transduced OECs showed no accumulation in major organs and were found at the injury site. After OEC transplantation, in the spinal cord tissues, the interleukin-1 receptor antagonist (IL-1Ra) was highly upregulated while many chemokines, including pro-inflammatory chemokines IL-1α, IL-1β were downregulated. *In vitro* studies confirmed that lipopolysaccharide (LPS) stimulus triggered OECs to secrete IL-1Ra. OECs significantly suppressed LPS-stimulated microglial activity, whereas IL-1Ra gene knockdown significantly reduced their ability to modulate microglial activity.

**Conclusion:** The OECs that reached the lesion site were activated by the release of pro-inflammatory cytokines from activated microglia in the lesion site and secreted IL-1Ra to reduce neuroinflammation. Intravenous transplantation of OECs has high therapeutic effectiveness for the treatment of SCI *via* the secretion of IL-1Ra to reduce neuroinflammation.

## Introduction

Spinal cord injury (SCI) not only causes disability for individuals, but also represents a burden to health-care systems 1. After the initial insult to the spinal cord, the injury triggers a cascade of pathological events 2, including neuroinflammation, which has been described as a pathological hallmark leading to further neuronal death. Therefore, neuroinflammatory reactions have been treated as a potential therapeutic target in SCI 3-6.

The neuroinflammatory responses following SCI are mediated by several pro-inflammatory or anti-inflammatory cytokines 7-9. Extensive data have shown that reactive astrocytes and microglia secrete pro-inflammatory cytokines and chemokines, such as interleukin (IL)-1, IL-6, and tumor necrosis factor (TNF)-α, which initiate the effective cascades. These cascades amplify the inflammatory responses and destroy the internal microenvironment, resulting in cell death and inhibition of axonal regeneration 10,11. Among these inflammatory mediators, the IL-1 family is particularly important for the ability to activate and regulate the inflammatory process 12-14. Evidence suggests that therapeutic approaches to inhibit the expression of pro-inflammatory cytokines or increase anti-inflammatory cytokines could modulate the localized microenvironment and enhance regeneration after SCI 15-17. As an effective intervention target, IL-1-blocking therapy using monoclonal antibody and the delivery of anti-inflammatory drugs could significantly ameliorate the effects of IL-1 in various animal models of neuroinflammatory diseases 18-21.

In the last few decades, cell therapy has been applied to treat SCI in animal experiments and clinical trials 22-25. Among these cells, olfactory ensheathing cells (OECs) have been regarded as one of the most promising therapeutic candidates. In a previous study, we found that intravenous OEC transplantation 1 day after SCI significantly improved locomotion and decreased inflammation in spinal cord sections 26. Thus, we speculate that the cell's function in regulating the inflammatory environment following SCI may play an essential role in its therapeutic effect. In this study, microglial activation was monitored *in vivo* and *ex vivo* after intravenous transplantation of OECs. And the cellular fate of OECs was tracked by reporter gene imaging. In order to explore possible mechanism, protein microarray was employed to analyze the proteins significantly differentially expressed in spinal cord after OEC transplantation. The result was further verified *in vitro*. Together, these data indicate that the major anti-inflammation mechanisms of OECs might be based on the modulation of microglial activity through IL-1Ra release.

## Results

### *In vivo* and *ex vivo* PET studies of neuroinflammation

Some of these results were reported in abstract form at the SNMMI 2020 Annual Meeting 27.

**Figure 1:**
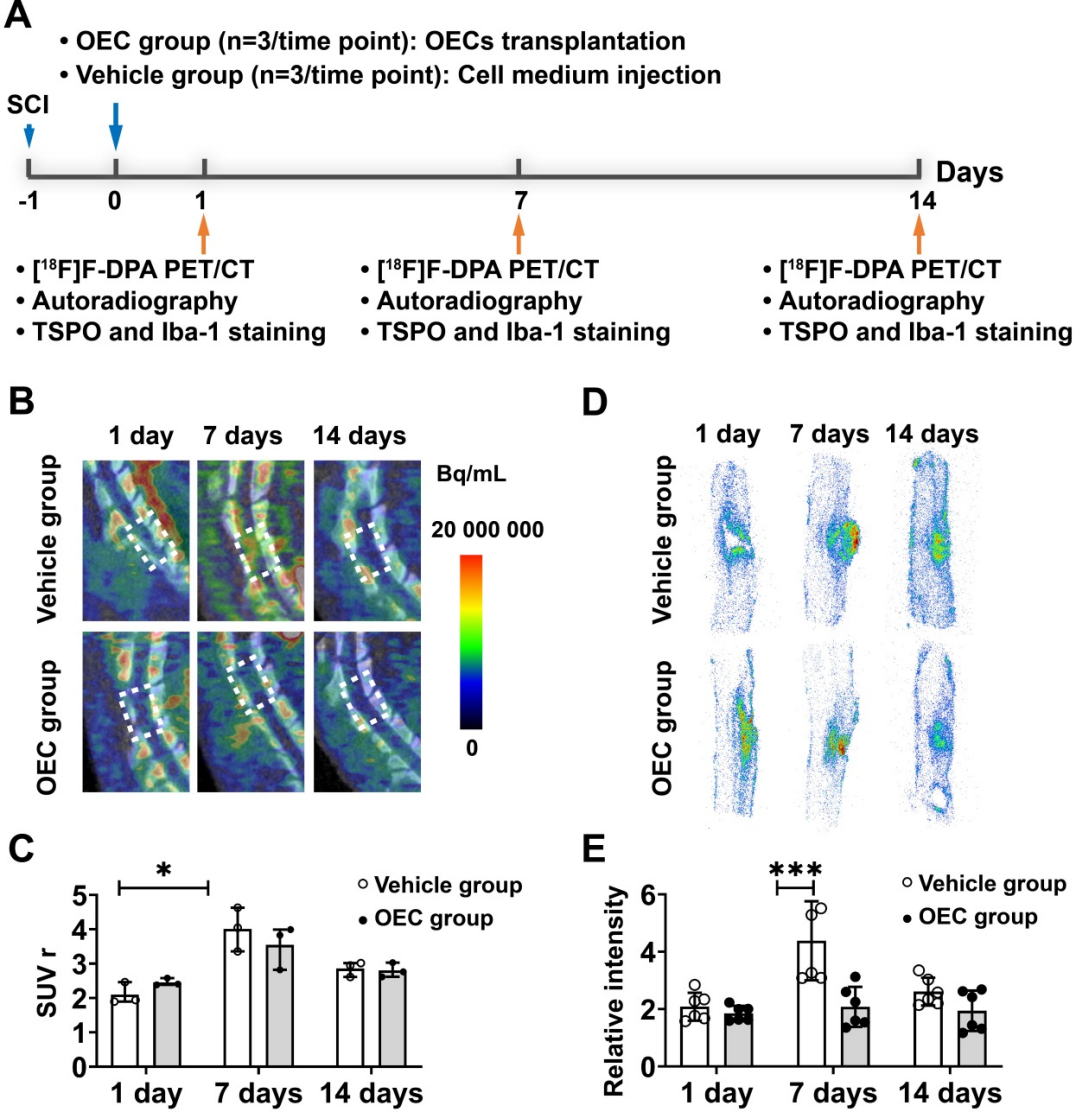
** [^18^F]F-DPA PET/CT imaging and spinal cord autoradiography. (A)** Design of the [^18^F]F-DPA study. **(B)** Representative [^18^F]F -DPA PET/CT images and standardized uptake ratio (SUV_r_) values. Images are summed over 40 - 60 min after injection. **(C)** Quantification of [^18^F]F-DPA uptake in the region of interest (ROI) (T11-T13) in the vehicle group and OEC group. **(D)** Representative [^18^F]F-DPA autoradiography images. **(E)** Quantitative analysis of the photostimulated luminescence (PSL/pixel) ratios in the ROI of the vehicle group and OEC group. *p < 0.05, **p < 0.01.

Figure 1A shows a diagram of the PET study using the TSPO binding radiotracer [^18^F]F-DPA to monitor microglial activation in the vehicle and OEC groups. Figure 1B shows representative PET/CT images at the injury site of the vehicle and OEC groups 1, 7, and 14 days after intravenous transplantation of vehicle or OECs. *In vivo* PET quantification using the standardized uptake value ratio (SUV_r_) showed a significant increase in [^18^F]F-DPA uptake in the vehicle group 7 days after injection (Figure 1C). The OEC group did not present differences in [^18^F]F-DPA uptake at each time point.

We also assessed [^18^F]F-DPA uptake by *ex vivo* autoradiography (ARG). Figure 1D shows representative ARG images at the injury site in the vehicle and OEC groups 1, 7, and 14 days after intravenous transplantation. After SCI, [^18^F]F-DPA accumulated around the injury site in both the OEC and vehicle groups, particularly in the glial scar. Seven days after transplantation, a larger glial scar formed at the injury site in the vehicle group compared to the OEC-treated group. In addition, the uptake of [^18^F]F-DPA was significantly higher in the vehicle group than in the OEC group (*p* = 0.025; Figure 1E).

**Figure 2:**
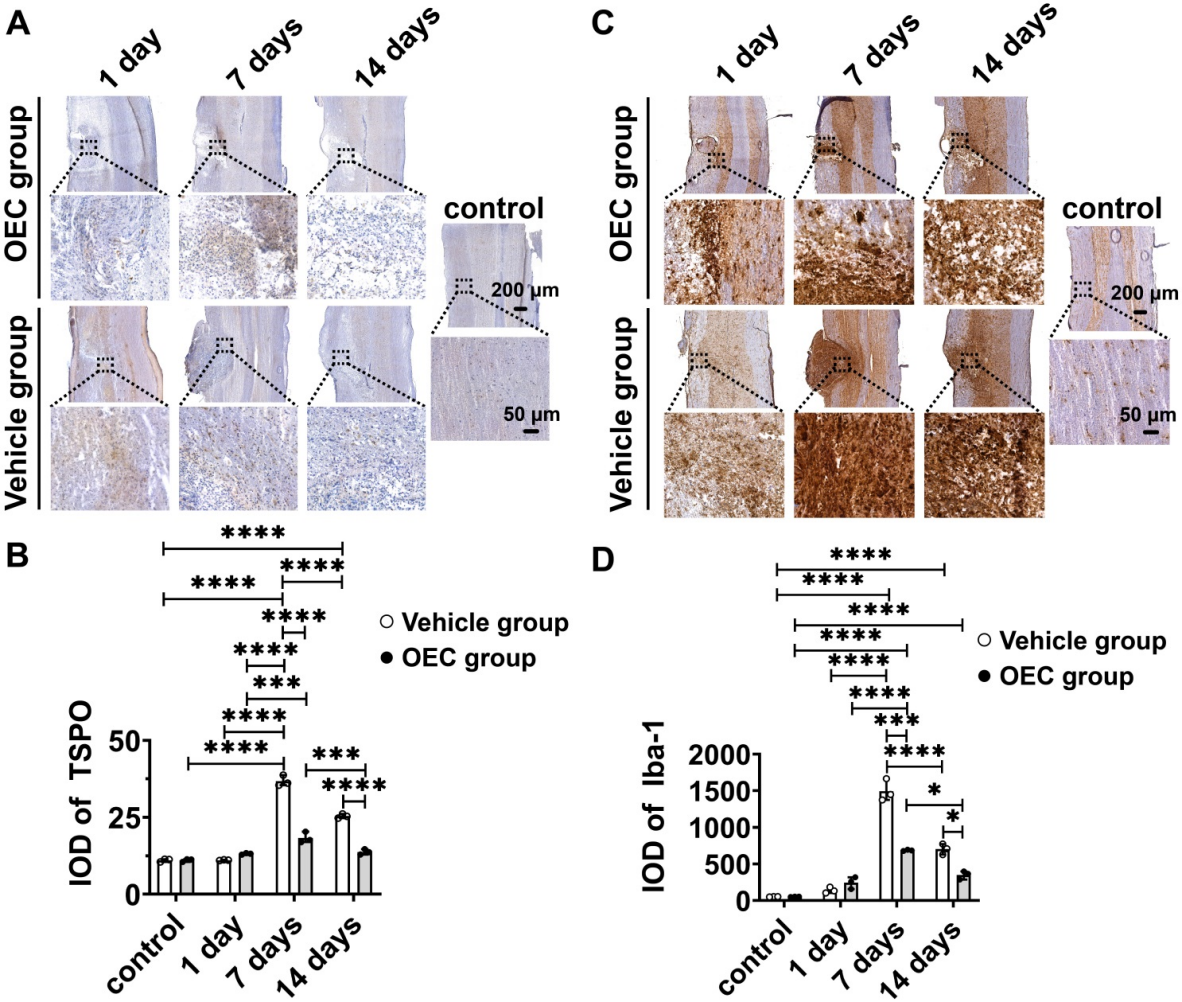
** Immunohistochemical staining of TSPO and Iba-1. (A)** TSPO immunostaining in the OEC group, vehicle group, and control group 1, 7, and 14 days after transplantation. **(B)** Quantification of TSPO expression (integrated optical density, IOD). **(C)** Iba-1 immunostaining in the OEC group, vehicle group, and control group 1, 7, and 14 days after transplantation. **(D)** Quantification of Iba-1 expression (IOD). *p < 0.05, ***p < 0.001, ****p < 0.0001; n = 5.

To confirm [^18^F]F-DPA binding, immunohistochemistry (IHC) with TSPO and Iba-1 specific antibodies was performed in the same spinal cord slices used for ARG. IHC showed increased expression of TSPO and Iba-1 in and around the injury site, especially in the glial scar (Figure 2A,C). The expression of TSPO and Iba-1 in the vehicle and OEC groups increased 7 and 14 days after transplantation compared to the control group and 1 day (*p* < 0.0001 for all comparisons). The expression of TSPO and Iba-1 was significantly higher in the vehicle group than in the OEC group at 7 days (*p* < 0.0001 and *p* = 0.008, respectively) and 14 days (*p* < 0.0001 and *p* = 0.028, respectively) after transplantation (Figure 2B,D).

### Tracking of OECs *in vivo* and *ex vivo*

**Figure 3:**
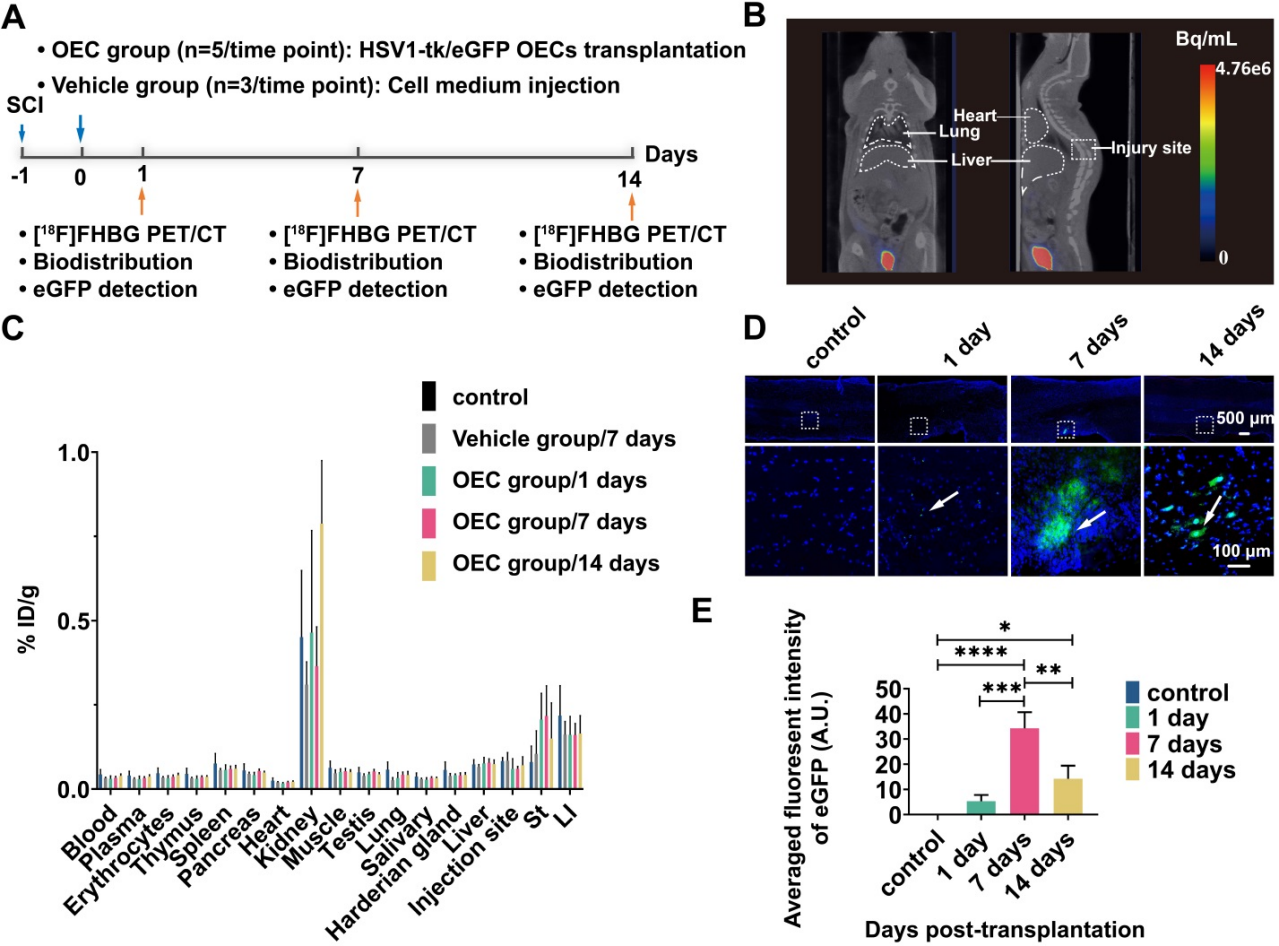
** [^18^F]FHBG PET/CT imaging and eGFP detection in the spinal cord. (A)** Design of the [^18^F]FHBG study. **(B)** Representative [^18^F]FHBG PET/CT images acquired 40 min after injection. **(C)** *Ex vivo* biodistribution of [^18^F]FHBG in the OEC group 1, 7, and 14 days after transplantation, the vehicle group 7 days after injection, and the control group. ST: stomach, LI: large intestine. **(D)** Detection of eGFP expression at the spinal cord injury site. **(E)** Quantification of the eGFP fluorescence intensity. **p < 0.01, ***p < 0.001.

The OECs underwent transduction with HSV1-39tk and eGFP as a means to track the transplanted OECs *in vivo* and *ex vivo* (Figure 3A). The radioligand [^18^F]FHBG, which specifically binds HSV1-39tk, was used to study the biodistribution of HSV1-39tk-expressing OECs. Figure 3B shows a representative [^18^F]FHBG PET/CT image 40 min after radiotracer injection. We found no differences between the PET/CT images 1, 7, or 14 days after HSV1-39tk-eGFP OEC transplantation. As shown by the [^18^F]FHBG biodistribution, we found no significant differences between the OEC group and vehicle group at any time point (Figure 3C). The highest accumulation of [^18^F]FHBG was in the kidneys and urine, indicating tracer excretion *via* the urinary tract. These results indicate that the transplanted OECs did not accumulate in any organs, especially the lung or liver.

The accumulation of transplanted OECs at the sites of SCI was further examined in the spinal cord slices (Figure 3D). Fluorescence imaging showed the presence of transplanted HSV1-39tk-eGFP OECs in the damaged areas of the spinal cord. As early as 1 day post-transplantation, some HSV1-39tk-eGFP OECs were already accumulated around the injury site. Seven days after transplantation, the number of HSV1-39tk-eGFP OECs was significantly increased compared to 1 and 14 days post-transplantation (*p* = 0.0002 and *p* = 0.002, respectively; Figure 3E).

### Protein microarray analysis of spinal cord tissue

**Figure 4:**
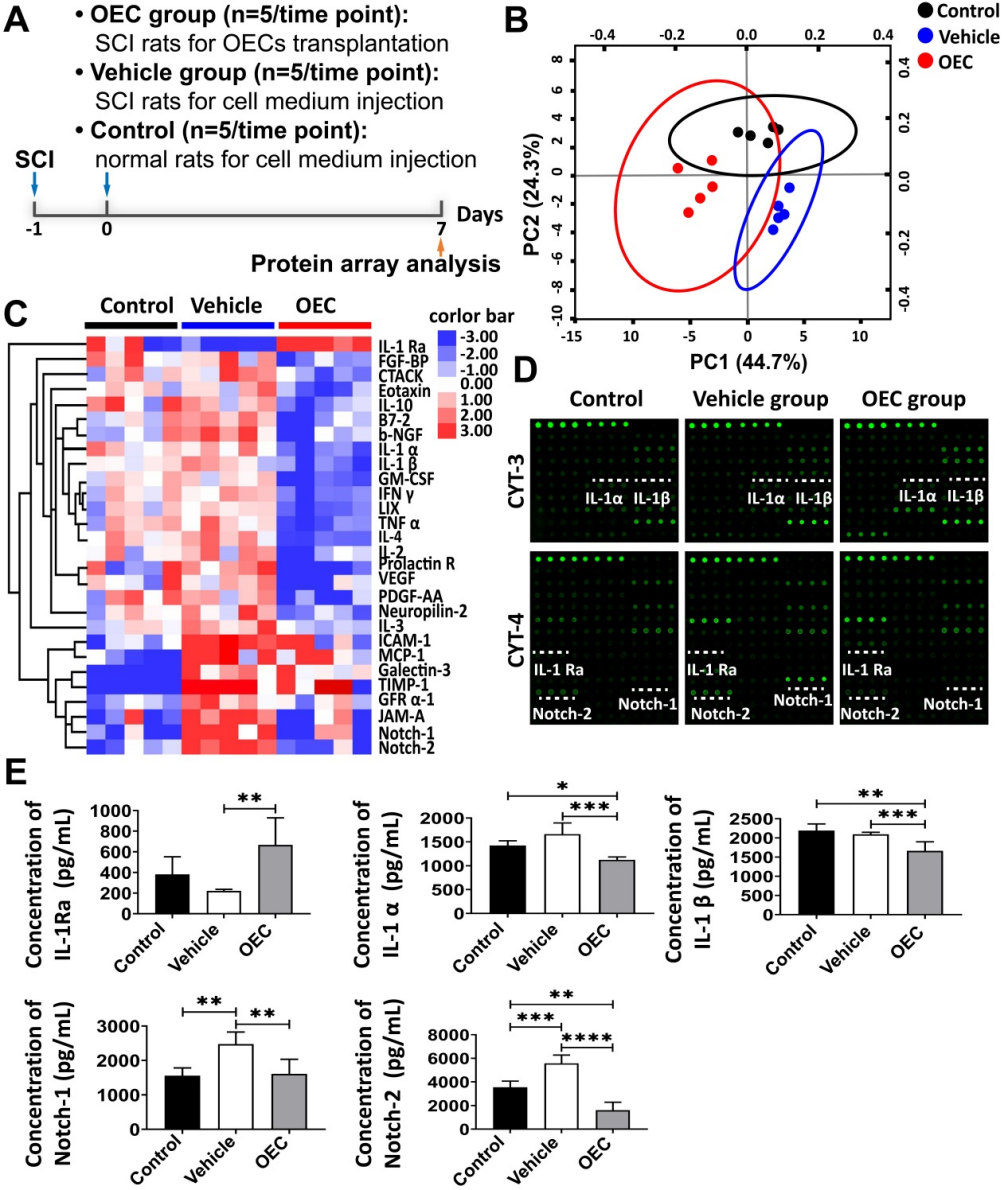
** Alternate protein profiles in the spinal cord induced by OEC transplantation. (A)** The study design for protein array analysis. **(B)** Principal component analysis among the OEC group, vehicle group, and control group. **(C)** Heat map presenting differentially expressed proteins in the OEC group, vehicle group, and control group. **(D)** Antibody array profiles among the three groups. IL-1α, IL-1β, IL-1Ra, Notch-1, and Notch-2 were detected with Raybio cytokine antibody array-3 and cytokine antibody array-4. Their location in the array is labeled by white underscores. **(E)** ELISA analysis of IL-1α, IL-1β, IL-1Ra, Notch-1, and Notch-2. *p < 0.05, **p < 0.01, ***p < 0.001.

To reveal the therapeutic mechanism of OECs in SCI treatment, we performed a protein microarray analysis of the spinal cord tissue from the OEC group, vehicle group, and control group 7 days post-transplantation (Figure 4A). Principal component analysis (PCA) of the microarray data showed three clearly separate groups (Figure 4B). A number of cytokines/chemokines, such as ICAM-1, MCP-1, TIMP-1, Galectin-3, GFR α-1, JAM-A, Notch-1, and Notch-2, in the vehicle group were significantly upregulated compared to the control group (Table S1 and Figure 4C-D). Comparing protein expression profiles between the vehicle group and the OEC group, the most conspicuous difference is that the OEC group exclusively induced significant upregulation of IL-1Ra. Among the cytokines expressed at different levels between the OEC group and the vehicle group, 22 were significantly downregulated, including IL-1α, IL-1β, Notch-1, and Notch-2 (*p* = 0.0003, *p* = 0.0047, *p* = 0.0046, and *p* < 0.0001, respectively; Table S2 and Figure 4C-D). Thus, we presumed that the anti-inflammatory effect of intravenous transplanted OECs for SCI might be associated with the secretion of IL-1Ra.

**Figure 5:**
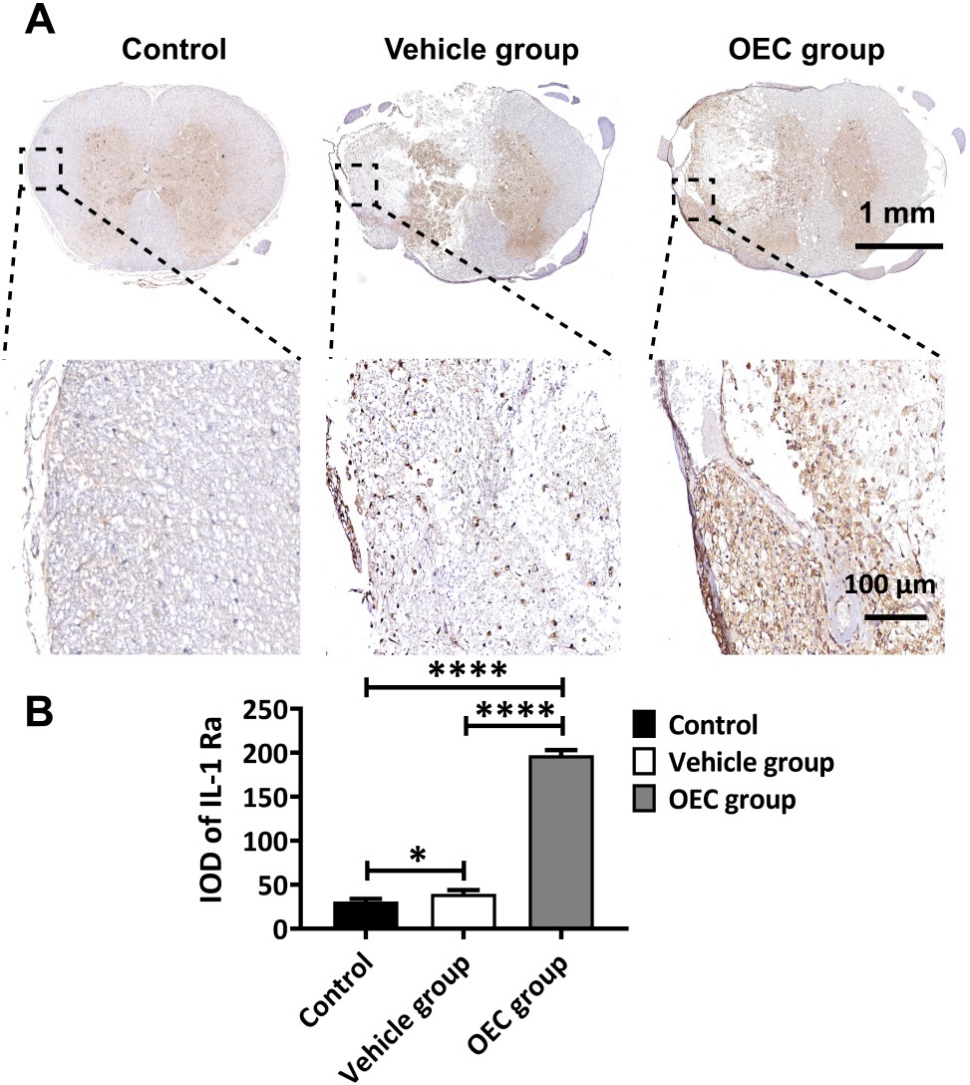
** Expression of IL-1Ra in the spinal cord after OEC transplantation. (A)** IL-1Ra immunostaining in the OEC group, vehicle group, and control group 7 days after transplantation. **(B)** Quantification of IL-1Ra expression (integrated optical density, IOD). *p < 0.05, ****p < 0.0001; n = 5.

The levels of IL-1α, IL-1β, IL-1Ra, Notch-1, and Notch-2 in the spinal cord 7 days after transplantation were also quantified by enzyme-linked immunosorbent assay (ELISA). Figure 4E shows the highly significant increase in IL-1Ra levels in the OEC group compared to vehicle (*p* = 0.0058). The levels of IL-1α and IL-β were significantly lower in the OEC group than in the control group (*p* = 0.0303 and *p* = 0.0059, respectively) and the vehicle group (*p* = 0.0047 and p < 0.0001, respectively). The levels of Notch-1 and Notch-2 were also significantly lower in the OEC group than the vehicle group (*p* = 0.0047 and *p* < 0.0001, respectively). The upregulation of IL-1Ra in the spinal cord tissues was validated by IHC (Figure 5), confirming the highly significant increase in IL-1 Ra in the OEC transplantation groups.

### OECs express IL-1Ra in response to inflammatory stimuli *in vitro*

**Figure 6:**
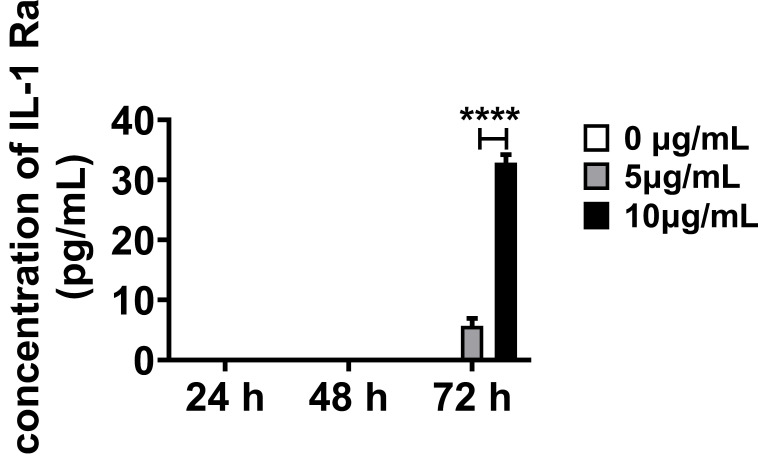
** Secretion of IL-1Ra by OECs under inflammatory stimulation.** The expression of IL-1Ra secreted by OECs under different concentrations and exposure time to lipopolysaccharide (LPS). ****p < 0.0001, n = 5.

We further investigated the anti-inflammatory response of OECs observed *in vivo* via* in vitro* inflammatory stimulation of OECs. Lipopolysaccharide (LPS) was selected as the stimulus according to the biological process in Gene Ontology (GO) enrichment analysis (Figure S2A, Table S3). And the cytotoxicity of LPS to OECs was analysis with MTT assay (Figure S4). IL-1Ra was under the limit of detection when the OECs were in normal cell culture medium (Figure 6). After treating OECs with LPS (5 and 10 μg/mL) for 72 h, the IL-1Ra levels were strongly elevated (*p* < 0.0001). Thus, these results demonstrate that OECs could secrete IL-1Ra in response to inflammatory stimuli.

**OECs release high amounts of IL-1Ra and suppress microglia activation**

To examine the inflammation-modulating potential of IL-1Ra released from OECs, the gene expression in OEC was knocked down by small interfering RNA (siRNA). OECs were transfected with siRNA 1, siRNA 2, or siRNA 3, with non-targeted control siRNAs (siRNA NC) used as a negative control and glyceraldehyde-3-phosphate dehydrogenase (GAPDH) as an internal reference. The results obtained from both Western blot and polymerase chain reaction (PCR) analyses indicated that the expression of IL-1Ra was knocked down best by siRNA 2 (Figure S3). Thus, siRNA 2-transfected OECs were used as the IL-1Ra knockdown OECs in subsequent experiments.

**Figure 7:**
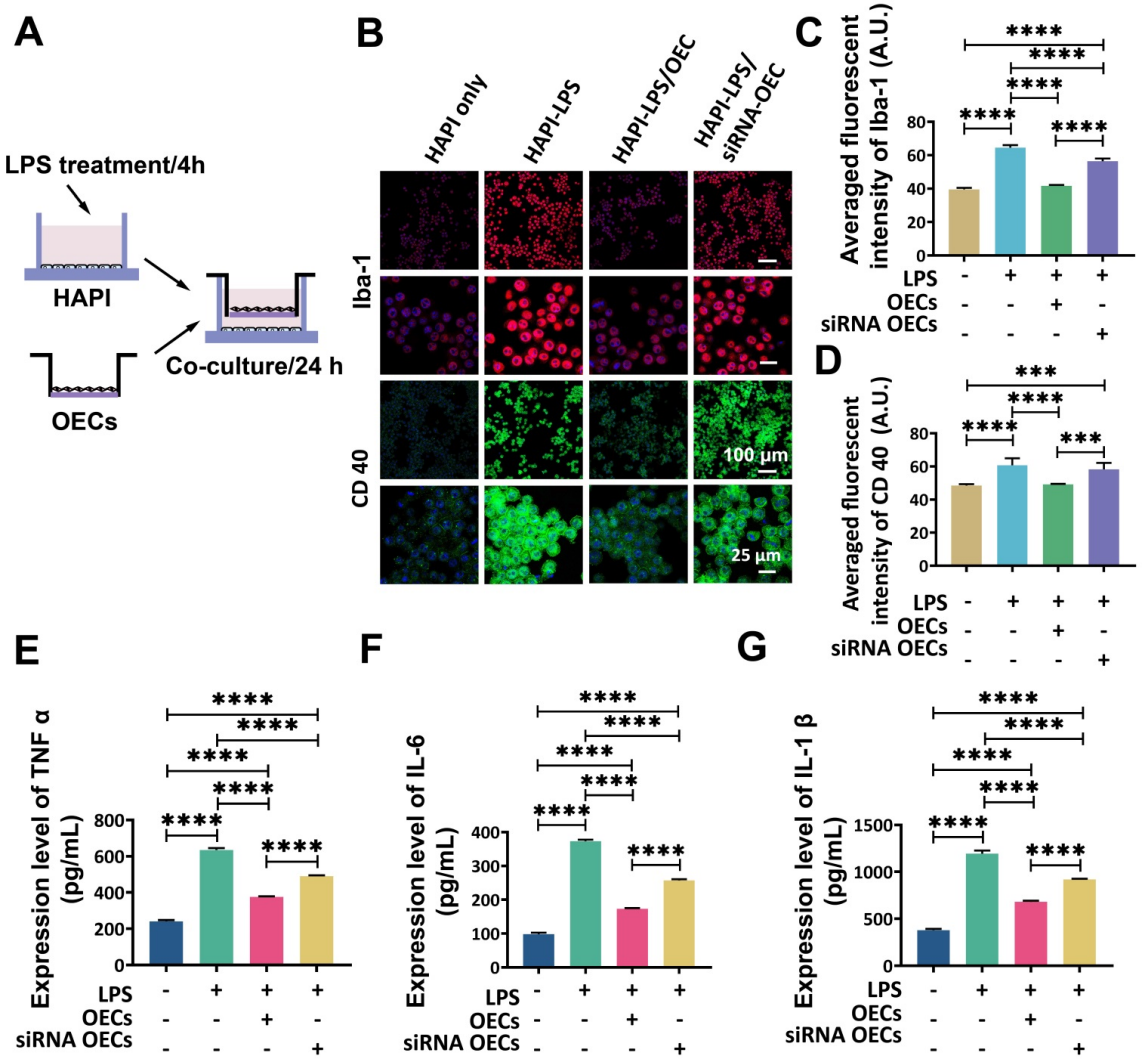
** OECs inhibited microglia activation *in vitro*. (A)** Study design for *in vitro* co-culture. **(B)** Immunofluorescent staining of microglia marker Iba-1 (red) and CD40 (green). **(C and D)** Quantification of the fluorescence intensity of Iba-1 (c) and CD 40 (d). **(E, F and G)** Expression of pro-inflammatory cytokines TNF α (e), IL-6 (f), and IL-1β (g) in the supernatant of HAPI cells after 24 h co-culture with HAPI microglia cells. ***p < 0.001, ****p < 0.0001; n = 5.

To further investigate the interaction between OECs and microglia, a transwell co-culture system was used. *In vitro* microglial activation was induced by LPS (Figure 7A). Highly aggressive proliferating immortalized (HAPI) microglial cells were treated with LPS (1 mg/mL) for 4 h 28 before co-culturing with OECs or IL-1Ra knockdown OECs in the transwell co-culture system for another 24 h. The results obtained from immunocytochemistry indicated that the LPS stimulation significantly activated HAPI microglial cells (Figure 7B). The averaged fluorescent intensity of microglial markers Iba-1 and CD40 was significantly increased (64.52 ± 1.37 *vs.* 39.48 ± 0.82, *p* < 0.0001 and 60.67 ± 3.85 *vs.* 48.48 ± 0.71, *p* < 0.0001, respectively). Following co-culture with OECs, the activation of HAPI microglial cells was reduced, as the averaged fluorescent intensity of Iba-1 and CD40 was remarkably lower than in the HAPI-LPS group (41.63 ± 0.49 *vs.* 64.52 ± 1.37, *p* < 0.0001 and 49.17 ± 0.26 *vs.* 60.67 ± 3.85, *p* = 0.0004, respectively). The averaged fluorescent intensity of Iba-1 was still significantly decreased in the HAPI-LPS/siRNA-OEC group compared to the HAPI-LPS group (64.52 ± 1.3756.377 ± 1.41 *vs.*, *p* < 0.0001). However, the averaged fluorescent of CD 40 in the HAPI-LPS/siRNA-OEC group was not significantly different from the HAPI-LPS group (58.30 ± 3.44 *vs.* 60.67 ± 3.85, p = 0.5847). The averaged fluorescent intensity of Iba-1 and CD40 was significantly higher in the HAPI-LPS/siRNA-OEC group than the HAPI-LPS/OEC group (56.377 ± 1.41 *vs.* 41.63 ± 0.49, *p* < 0.0001 and 58.30 ± 3.44 *vs.* 49.17 ± 0.26, *p* = 0.0008, respectively). These results indicate that the ability to reduce the activity of HAPI microglia under inflammatory stimulation decreased in the siRNA-OECs (Figure 7C-D).

The expression of pro-inflammatory cytokines TNF-α, IL-6, and IL-1β in the supernatants of HAPI cells was significantly enhanced after LPS stimulation (240.89 ± 6.05 *vs.* 635.26 ± 8.06, *p* < 0.0001; 98.65 ± 3.30 *vs.* 373.2 9± 3.60, *p* < 0.0001; and 379.06 ± 11.54 *vs.* 1193.74 ± 26.81, *p* < 0.0001, respectively; Figure 7E, 7F,7G). After OEC or siRNA-OEC treatment, the expression of pro-inflammatory cytokines released from microglia was significantly reduced (*p* < 0.0001 for both), but the expression of TNF-α, IL-6, and IL-1 β was significantly higher in the HAPI-LPS/siNRA-OEC group than the HAPI-LPS/OEC group (491.07 ± 2.99 *vs.* 375.09 ± 3.06, *p* < 0.0001; 257.09 ± 3.10 *vs.* 173.13 ± 1.88, *p* < 0.0001; and 919.27 ± 6.44 *vs.* 680.91 ± 9.48, *p* < 0.0001, respectively).

**Figure 8:**
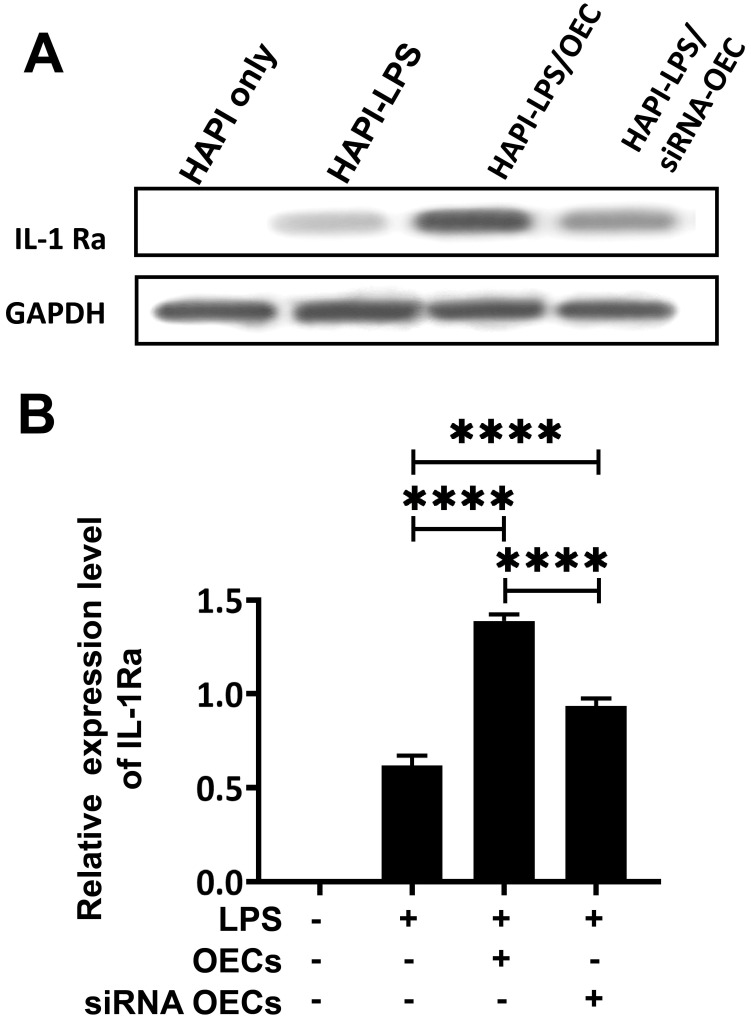
** Co-culture with activated microglia increases the secretion of IL-1Ra from OECs. (A)** Western blot analysis of IL-1Ra expression in the supernatant after co-culture with HAPI microglia. **(B)** IL-1Ra expression in the co-culture supernatant. ****p < 0.0001, n = 5.

Western blot analysis showed that the HAPI microglia could express a basal level of IL-1Ra following LPS stimulation. After co-culturing OECs or siNRA-OECs with LPS-stimulated HAPI microglia, the expression of IL-1Ra was significantly upregulated in both the HAPI-LPS/OEC group and the HAPI-LPS/siNRA-OEC group (*p* < 0.0001 for both). The relative expression of IL-1Ra was significantly higher in the HAPI-LPS/OEC group than the HAPI-LPS/siNRA-OEC group (*p* < 0.0001; Figure 8).

## Discussion

Microglial activation and inflammation are highest in the subacute phase of SCI 29. Intravenous OEC transplantation 1 day after SCI significantly improves functional outcomes, and effectively decreases inflammation 26. In the present study, we explored the anti-inflammatory roles of OECs, revealing for the first time the possible therapeutic mechanisms underlying intravenous transplantation of OECs. The major mechanism may be that the OECs that reach the lesion site, stimulated by the release of pro-inflammatory factors from activated microglia in the lesion site, secrete IL-1Ra to reduce microglial activation and pro-inflammatory factors secreted by microglia.

The *in vivo* PET/CT study and spinal cord ARG analysis showed that the inflammation, which extensively increased without treatment, was significantly decreased 7 days after OEC transplantation. It is well described that the formation of the glial scar is a response to injury, and although the glial scar can isolate the damage, it also inhibits functional recovery 30. Modulation of the inflammatory response in the acute and subacute phase of SCI is important for recovery and formation of the glial scar 31. In the current study, the area of the glial scar was dramatically decreased in the OEC treatment group compared to the untreated group. In addition, the OEC-treated rats with much smaller glial scars, and the previous study showed that these rats recovered better than the vehicle group. Our results suggest that the transplanted OECs reduce the formation of the glial scar by modulating activated microglia in the acute and subacute phase of SCI, which may improve recovery.

To further investigate the anti-inflammatory mechanism of OECs, we analyzed the expression profiles of inflammation related chemokines and cytokines in the injured spinal cord 7 days after transplantation. The results revealed that intravenous transplantation of OECs significantly increased IL-1Ra expression in the injured spinal cord compared to the normal group and vehicle group, which was also confirmed by IHC. IL-1Ra is a competitive inhibitor for the binding of IL-1 to its receptor 32 and reduces the synthesis and release of pro-inflammatory cytokines 33. Previous *in vivo* and *in vitro* studies showed that IL-1Ra could reduce the inflammatory reaction and enhance neuronal preservation after SCI 34-36. We also found that IL-1α and IL-1β, which belong to the IL-1 family and exert strong inflammatory activities, were downregulated after OEC transplantation in the injured spinal cord. A previous study with an SCI model combining minocycline treatment and OEC transplantation described a decrease in TNF-α and IL-1β 5 weeks after injury 37.

LPS is a Toll-like receptor that elicits a strong response by promoting the secretion of pro-inflammatory cytokines 38,39. The neuroinflammatory role of OECs can be activated in response to LPS 40,41. Moreover, the GO analysis of the microarray showed that the response to LPS is specifically enriched. Therefore, LPS was used in this study as an inflammatory stimulus. When exposed to LPS, OECs released IL-1Ra in a time-dependent and dose-dependent manner, but no IL-1Ra was detected in the supernatants from OECs cultured under normal conditions. This further suggests that OECs could be activated in response to the inflammatory microenvironment at the epicenter of SCI and subsequently release IL-1Ra to suppress microglial activation. The modulation of microglial activation by OECs was further investigated *in vitro* and showed that the main anti-inflammation mechanisms might be based on the modulation of microglial activity through IL-1Ra release.

Although the efficacy of intravenous OEC transplantation has been observed, their fate has not yet been demonstrated. The safety of cell transplantation also needs to be evaluated by monitoring the viability, biodistribution, and trafficking of the transplanted cells. Previous studies have shown that intraspinal or intrathecal transplantation of OECs has positive results in the treatment of SCI 42,43. However, these methods of transplantation carry some degree of risk for additional neurological damage to the injured spinal cord. As a convenient and non-invasive route for cell delivery in a clinical setting, intravenous transplantation has attracted much interest from clinicians and researchers regarding SCI. In this study, OEC was engineered to express the HSV1-39tk-eGFP reporter gene and monitored by [^18^F]FHBG PET imaging and fluorescence microscopy after intravenous transplantation. The major concern with intravenously injecting cells is that the cells could form clots in the lungs or liver, or tumorigenicity after incorporation into the tissues. [^18^F]FHBG PET/CT and the biodistribution results showed that the intravenously injected OECs did not clot, accumulate, or proliferate in the lungs or liver. Moreover, HSV1-39tk-eGFP-transduced OECs were found at the injury sites. Thus, a small fraction of the transplanted OECs could reach the injury site. Combined with the previous study^24^, these results indicate that OECs translocated to the injury site through the disrupted blood-spinal cord barrier. The significant decreased number of transplanted OECs at 14 days post transplantation showed low risk of tumorigenicity. OECs are not stem cells and do not have the ability to differentiate. We speculate that the intravenous transplantation of OECs has preliminarily been shown to be safe. Moreover, considering the narrow time-window for blood-spinal cord barrier disruption and the highly active inflammation in the acute and subacute phases of SCI, we recommend a time-window for intravenous transplantation of OECs in the acute and subacute stages of SCI.

The intravenous transplantation of OECs suppressed the neuroinflammatory responses in a hemisection rat model of SCI and reduced the glial scar. In addition, we demonstrated that OECs produce IL-1Ra when activated by the inflammatory microenvironment of SCI. The intravenous transplantation of OECs was determined to be safe. These results provide a scientific basis for the intravenous transplantation of OECs in clinical applications.

## Materials and Methods

### Animals

All experimental procedures were approved by the Regional State Administrative Agency for Southern Finland (ESAVI/10106/2018) and Ningxia Medical University Animal Ethics Committee (2018-054). A total number of 72 male Sprague-Dawley rats weighing 270-800 g were used as follows: [^18^F]F-DPA study, n = 18, from Janvier Labs (Le Genest Saint Isle, France); [^18^F]FHBG study, n = 24, from the Central Animal Laboratory (University of Turku); protein microarray study, n = 15, from the Experiment Animal Center of Ningxia Medical University (Yinchuan, China); IL-1Ra IHC study, n = 15, from the Experiment Animal Center of Ningxia Medical University (Yinchuan, China).

All rats were group-housed after surgery under consistent temperature (21 ± 3 °C) and humidity (55 ± 15%) conditions with a 12-h light/dark cycle. The rats were fed soy-free chow (RM3 (E) soya-free, 801710, Special Diets Service) and water ad libitum.

### Spinal cord injury model

To create a SCI model, rats were anesthetized with isoflurane (4-5% induction, 2% maintenance) and body temperature maintained using a heating pad. The skin over the upper thoracic area was shaved and cleaned with betadine solution. The skin was incised and the connective and muscle tissue bluntly dissected to expose the thoracic tenth (T12) vertebral body, taking care not to damage the spinal dura during removal of the dorsal lamina. A lateral hemisection was performed at T12; initially, an angled needle punctured the spinal cord dorsoventrally at the midline, avoiding the dorsal spinal artery, and then pulled to cut the left half of the spinal cord. This operation was repeated three times to ensure completeness of the hemisection 24. For pain management, Rimadyl (5 mg/kg, Zoetis, Florham Park, NJ) and Temgesic (0.3 mg/mL, Intervet International, Boxmer, the Netherlands) was administered subcutaneously before surgery and every 8 h for 3 consecutive days after establishing the SCI model.

### OEC culture

OECs were isolated from the olfactory bulbs of newborn male Sprague-Dawley rats (6-7 weeks old, Central Animal Laboratory, University of Turku) as described previously 44. The morphology and immunostaining characteristics of the purified OECs were determined in our previous study 45. Purified OECs were passaged three to four times (6-8 days *in vitro*) before transplantation. OEC transplantation was performed 1 day post-injury. The rats received the transplantation of OECs (1 × 10^6^/mL) or cell culture medium *via* a tail vein (1mL per rat).

### [^18^F]F-DPA PET imaging

The synthesis of [^18^F]F-DPA was synthesized as described previously 46. Rats were anesthetized with 1.5-2.5% isoflurane/oxygen on the heating bed for micro PET/CT (Inveon multimodality PET/CT, Siemens Medical Solutions, fuji Knoxville, TN, USA) and a few drops of Oftagel (25 mg/g; Santen, Tampere, Finland) were applied to prevent eye dryness. CT was performed for 10 min as an attenuation and anatomical reference. A 60-min dynamic PET scan (30 × 10 s, 15 × 60 s, 4 × 300 s, and 2 × 600 s) was acquired after intravenous injection with [^18^F]F-DPA (22.4 ± 4 MBq). To analyze the uptake of [^18^F]F-DPA (Inveon Research Workplace 3.0, Siemens medical Solutions, Knoxville, TN, USA), images were summed over 40-60 min after tracer injection. The uptake of [^18^F]F-DPA was quantitatively assessed using SUV_r_, which is the ratio between the mean SUV at the injury region (T11-T13) and the mean SUV at a reference region (C2-C4).

### Spinal cord autoradiography of [^18^F]F-DPA

Following PET imaging, the rats were sacrificed by transcardial perfusion with saline under deep anesthesia. The spinal cord at T11-13 was dissected and frozen by immersion in dry ice-chilled isopentane (Sigma-Aldrich). Longitudinal spinal cord sections (20 μm) were obtained on a cryomicrotome (Leica CM3050S, Germany) and collected on a glass slide (Superfrost Ultra Plus, Thermo Fisher, USA). The slides were exposed to an imaging plate (Fuji BAS-TR2025, Fuji Photo Film Co., Tokyo, Japan) for autoradiography, with an exposure time of approximately two half-lives. The digital images were obtained by a BAS-5000 scanner (Fuji Photo Film Co., Tokyo, Japan) with a resolution of 25 µm and analyzed by AIDA software (Version 4.5, Raytest, Straubenhardt, Germany). For each rat, 3 sections were selected for analysis. At each section, the regions of interest (ROIs) were drawn manually with a length of 8 mm with the injury site at the center. The reference region was selected outside the ROI. The photostimulated luminescence (PSL) ratios were calculated as: (PSL/pixel in the ROI - background PSL/pixel) / (PSL/pixel in the reference ROI - background PSL/pixel). Three sections were analyzed for each rat.

### Immunohistochemistry

Seven days after OEC transplantation, the animals were anesthetized with 60 mg/kg sodium pentobarbital intraperitoneally and perfused with cold saline. After removing the vertebra, the spinal cord was isolated and fixed in 10% (v/v) neutral phosphate buffered formalin solution. For IHC, formalin-fixed, paraffin-embedded spinal cord sections were dewaxed and rehydrated for antigen retrieval. The sections were incubated overnight at 4 °C with the following primary antibodies: anti-Iba-1 antibody (1:50, ab139590, Abcam, Cambridge, MA), anti-PBR antibody (1:10,000, ab109497, Abcam, Cambridge, MA), and anti-IL-1Ra antibody (1:200, ab217939, Abcam, Cambridge, MA). The sections were then incubated with biotinylated goat anti-rabbit IgG for 20 min at room temperature, followed by streptavidin-peroxidase (all from Santa Cruz Biotechnology). The immunohistochemistry images were obtained under a light microscope (Leica DM4000M, Germany).

### Generation of stable HSV1-39tk-eGFP-expressing OECs

The lentivirus overexpressing HSV1-39tk-eGFP was designed and produced by GeneChem (Shanghai, China). For transduction, 5 × 10^5^ OECs were seeded in 6-well culture dishes and allowed to adhere overnight. The next day, OECs were transfected with HSV1-39tk-eGFP-expressing lentivirus, which was diluted with Enhanced Infection Solution (GeneChem, Shanghai, China) at a multiplicity of infection (MOI) of 10 according to the manufacturer's instructions. When assessing the transduction efficiency, the eGFP expression was observed with an Olympus CX41 Microscope (Olympus Corporation, Tokyo, Japan). Immunocytofluorescence was also performed to validate HSV1-sr 39TK expression in the stably transfected OEC population (Figure S5).

### [^18^F]FHBG synthesis and PET imaging

[^18^F]FHBG was prepared using the one-pot synthesis method developed from previously published methods 47, 48. Briefly, tosyl-FHBG precursor (2960, ABX, Radeberg, Germany) was radiolabeled using [^18^F]KF and Kryptofix 222 complex in DMSO (Sigma-Aldrich), and the protecting groups were hydrolyzed by 1 M HCl. The reaction mixture was neutralized and the final product purified by HPLC. The radiochemical yield was 6 ± 2% (decay corrected) with a radiochemical purity of 95 ± 3% and molar activity > 48.9 GBq/μmol at the end of the synthesis. The [^18^F]FHBG solution with 50 mM ammonium acetate, 0.9% NaCl, and 7% ethanol was used for animal experiments.

Rats were anesthetized with 1.5 - 2.5% isoflurane/oxygen and [^18^F]FHBG (9.0 ± 2.3 MBq) injected intravenously. Static PET imaging data were acquired over 20 min on micro PET/CT scanners (β- and X-CUBE, MOLECUBES, Gent, Belgium) starting 40 min after injection.

### *Ex vivo* biodistribution of [^18^F]FHBG

After [^18^F]FHBG PET imaging (i.e., 60 min after tracer injection), the rats were sacrificed by cardiac puncture under deep anesthesia. Blood was collected in heparin tubes. To eliminate the rest of the blood, saline was perfused transcardially. The organs were collected and radioactivity measured in a gamma counter (Wizard^2^ 3 × 3”, PerkinElmer, Turku, Finland) and weighted. The decay-corrected radioactivity is expressed as the percentage of injected dose per gram of tissue (%ID/g).

### Immunocytofluorescence

HSV1-tk immunocytofluorescence in OECs was performed 96 h post-transduction. OECs were fixed in 4% paraformaldehyde (PFA) for 15 min. The samples were rinsed three times with PBS, permeabilized for 10 min in 0.1% (v/v) Triton X-100 in PBS, blocked using 1% (w/v) bovine serum albumin (BSA) in PBS for 30 min, and incubated with rabbit TK1 polyclonal antibody (1:100, 15691-1-AP, Proteintech, Wuhan, China) overnight at 4 °C. Slides were washed in PBS three times for 10 min before adding rhodamine (TRITC)-conjugated goat anti-rabbit IgG (1:100, SA00007-2, Proteintech, Wuhan, China) and incubating for 1 h at room temperature. Slides were washed and coverslips mounted using mounting medium with DAPI (Beyotime Institute of Biotechnology, Shanghai, China). Images were acquired at room temperature using an Olympus FV1000 spectral confocal microscope (Olympus Corporation, Tokyo, Japan) with an UPLFLN 40XO objective lens (NA 1.30) and Olympus FLOWVIEW acquisition software.

### Antibody array assay of spinal cord tissue

For protein samples from the spinal cord tissues, a 1-cm segment of the spinal cord centered at the injury site was harvested from the sacrificed animals. The protein expression levels in the localized spinal cord were measured with rat cytokine antibody arrays (RayBio Rat Cytokine Antibody Array 3 and 4, glass series; RayBio Growth Factor Antibody Array, membrane series; RayBiotech, Norcross, GA, United States), which simultaneously detects 67 cytokines (Table S4), according to the manufacturer's instructions. Briefly, protein extracts were diluted to 500 mg/mL with blocking buffer and added to the array pools printed with 75 corresponding anti-cytokine antibodies for overnight incubation. After washing, a biotin-conjugated anti-cytokine mix was incubated with the pools for 2 h. Finally, the glass series arrays were performed with Cy3-conjugated streptavidin, and the membrane series arrays were incubated with HRP-conjugated streptavidin for another 2 h. After the experimental procedure, the glass slides were scanned to detect fluorescent signals using an InnoScan 300 Microarray Scanner (Innopsys, France). The membrane arrays were exposed after treatment with HRP catalyzing chemiluminescent solution using an Image Quant LAS4000 Scanner (GE Healthcare, Waukesha, WI, United States). The signals were read and normalized using an internal positive control and the RayBiotech analysis tool, which is specifically designed to analyze Rat Cytokine Antibody Array 3 and 4 and Growth Factor Antibody Array.

To determine the main effects of OEC treatment on SCI, the protein expression profiles of OEC and sham rats were compared to the vehicle rats, and limit values were set at *p* < 0.05 and fold change (FC) ≤ 0.83 or ≥ 1.2 to select the differentially expressed proteins. To investigate the biological importance underlying numerous proteins, the proteins with significant differential expression in the spinal cord samples were analyzed by bioinformatics (http://www.expasy.org/vg/index/protein). Gene Ontology (GO) and Kyoto Encyclopedia of Genes and Genomes (KEGG) enrichment analysis of these protein factors was conducted to screen the functions of the candidate cytokines associated with the inflammation modulating property of OECs. The results are given in Figure S1 and Figure S2 and Tables S5-7. *P* < 0.05 and protein count ≥ 2 were set as the cut‑offs.

To validate the results obtained from the antibody array, ELISA kits (RayBiotech, Norcross, GA, United States) were used to measure IL-1α, IL-β, and IL-1Ra expression levels according to the manufacturer's instructions. Briefly, protein extracts from spinal cord samples or supernatants were incubated in plates coated with capture antibody overnight at 4 °C. The plates were washed and a biotin-conjugated detection antibody added to the plates for incubation for 2 h at room temperature to combine with corresponding proteins. HRP-conjugated streptavidin was added to the plates and allowed to incubate for 45 min. Tetramethylbenzidine dihydrochloride (TMB) reagent was added and allowed to incubate for 30 min before the reaction was stopped with sulfuric acid. Immediately, the optical density was measured by an ELx800NB microplate reader (BioTek, Winooski, CT, United States) at a wavelength of 450 nm.

### Stimulation of OECs with LPS *in vitro*

Purified OECs were re-plated onto a 24-well petri dish at a final density of 1×10^5^ cells/well. LPS (0, 5, or 10 μg/mL, L2630, Sigma-Aldrich, Germany) were used as an inflammatory stimulus and added to OECs. The supernatants were collected and transferred at 24, 48, or 72 h to fresh tubes and stored at -80 °C. The supernatants were collected for detection of IL-1Ra using the IL-Ra ELISA kit (RayBiotech, Norcross, GA, United States) according to the manufacturer's protocol.

### siRNA knockdown of IL-1Ra in OECs

For IL-1Ra knockdown, OECs (5 × 10^5^ cells) were seeded on a 6-well culture plate. siRNAs (Santa Cruz Biotechnology, Santa Cruz-CA, USA) were used to treat the OECs according to the manufacturer's instructions (Table S8). Non-targeting control siRNAs (Santa Cruz Biotechnology, Santa Cruz-CA, USA) were used as negative controls. The efficiency of siRNA knockdown was confirmed by Western blot and qRT-PCR analysis.

### qRT-PCR analysis

The qRT-PCR analysis was performed 48 h post-siRNA transfection. Total RNA was extracted using the TRIzol reagent (15596026, Thermo Fisher Scientific, Waltham, MA, USA) according to the manufacturer's protocol. The quantity and quality of total RNA were measured using a NanoDrop 2000 spectrophotometer (Thermo, Wilmington, DE, USA) and RNA integrity determined by agarose gel electrophoresis. cDNA was generated using SuperScript^TM^ III Reverse Transcriptase (18080093, Invitrogen, Carlsbad, CA). The relative expression of IL-1Ra was determined using FastStart Universal SYBR Green Master (Rox) (Roche, Inc., Basel, Switzerland) with specific primers (GenerayBiotech, Shanghai, China), which are listed in Table S9. PCR was performed in triplicate using the following temperature profile: denaturation at 95 °C for 3 min followed by amplification in 40 cycles at 95 °C for 45 s and 60 °C for 20 min. GAPDH was used as an internal reference.

### Western blot analysis

After LPS (10 μg/mL) stimulation for 72 h, OECs and siRNA-treated OECs were scraped off and lysed in RIPA lysis buffer (Beyotime, Shanghai, China). The BCA protein assay kit (Beyotime, Shanghai, China) was used to measure the protein concentration. Briefly, 20 μg of total protein was separated by 10% SDS-PAGE and transferred to PVDF membranes (BioSciences, Pollards Wood, UK) at 300 mA for 40 min in an ice water mixture. The membranes were incubated overnight at 4 °C with the following primary antibodies: rabbit anti-GAPDH (1:1000, ab9485, Abcam) and rat anti-IL-1Ra (1:100, ab124962, Abcam). Subsequently, the membranes were blocked for 1 h at room temperature with 5% milk dissolved in TBST. The secondary HRP-conjugated antibodies were as follows: goat anti-rabbit IgG H&L (1:5000, ab6721, Abcam, Cambridge, UK). After washing three times with TBST, an ECL kit (32109, Thermo Fisher Scientific, Waltham, MA, USA) was used to detect the immunoactivity.

### Co-culture of OECs or siRNA OECs with HAPI microglial cells

The co-culture experimental design is shown in Figure 7A. HAPI rat microglial cells (SCC103, Sigma-Aldrich, Germany) were seeded in 6-well plates (1×10^5^ cells each). To induce HAPI cell reactivity, we used 1 mg/mL of LPS diluted in complete medium for 4 h. OECs or siRNA OECs were seeded (1×10^5^ cells each) in Transwell plate inserts (Millipore). HAPI cells were co-cultured with the OECs or siRNA OECs for another 24 h. To explore their roles in modulating inflammation, the culture supernatants were used for ELISA of pro-inflammatory cytokines, including TNFα (ab100785, Abcam, Cambridge, UK), IL-1β (ab100767, Abcam, Cambridge, UK), and IL-6 (ab100772, Abcam, Cambridge, UK).

### Immunofluorescent staining of microglial markers

HAPI microglia cells were fixed in 4% PFA for 10 min and rinsed with PBS three times. The samples were permeabilized with 0.03% Triton X-100 for 10 min, and then blocked using 1% (w/v) BSA in PBS for 30 min and incubated with primary antibodies CD40 (1:10, sc-514493, Santa Cruz Biotechnology, Santa Cruz-CA, USA) and Iba-1 (1:200, ab178846, Abcam) at 4 °C overnight. After washing off the primary antibody with PBS, secondary antibodies Alexa Fluor® 488 AffiniPure Goat Anti-Mouse IgG (H+L) (1:100, 115-545-003, Jackson ImmunoResearch, West Grove, PA, USA) and Alexa Fluor® 594 AffiniPure Donkey Anti-Rabbit IgG (H+L) (1:100, 711-585-152, Jackson ImmunoResearch, West Grove, PA, USA) were applied at 37 °C in the dark for 30 min. DAPI (C1002, Beyotime, Shanghai, China) was used to counterstain nuclei for 10 min in the dark at room temperature. Images were acquired at room temperature with an Olympus FV1000 spectral confocal microscope (Olympus Corporation, Tokyo, Japan) with an UPLFLN 40× objective lens (NA 1.30) and Olympus FLOWVIEW acquisition software. To quantify fluorescence, the fluorescence intensity under different transfection conditions were analyzed by Image J software (http://rsb.info.nih.gov/ij).

### Statistics

All data were statistically analyzed by repeated measures ANOVA followed by Dunnett's post-hoc test using GraphPad Prism 7.0 (GraphPad Software, La Jolla, CA, USA) and are presented as mean ± standard deviation (SD). Differences were considered significant if two-sided p-values were less than 0.05. In addition, fold change was given to indicate the relative expression levels of cytokines. The sample size was calculated based on the protein data for the antibody array altered in SCI and OEC-treated groups from the pilot study using Empower Stats software with two-tailed tests.

## Supplementary Material

Supplementary figures and tables.

## Abbreviations

OECs: olfactory ensheathing cells
SCI: spinal cord injury
IL-1Ra: Interleukin-1 receptor antagonist
LPS: Lipopolysaccharide
TNF-α: Tumor necrosis factor
HSV1-39tk: Herpes simplex virus type 1 thymidine kinase mutant
eGFP: Enhanced Green fluorescent protein
GO: Gene Ontology
KEGG: Kyoto Encyclopedia of Genes and Genomes
ARG: Autoradiography
IHC: immunohistochemistry
qPCR: quantitative real-time PCR
