# Supplementary material for: Intravenous transplantation of olfactory ensheathing cells reduces neuroinflammation after spinal cord injury via interleukin-1 receptor antagonist
Source: Theranostics. 2021 Jan 1;11(3):1147–61. doi: 10.7150/thno.52197 (PMC7738890; doi:10.7150/thno.52197)
Supplement: Supplementary file 1 — Supplementary figures and tables. [file thnov11p1147s1.pdf]

Supplementary Information for

Intravenous transplantation of olfactory ensheathing cells reduces neuroinflammation after spinal cord injury via interleukin-1 receptor antagonist

Lijian Zhang[1,2], Xiaoqing Zhuang[3, *], Päivi Kotitalo[4,5], Thomas Keller[6], Anna Krzyczmonik[6], Merja Haaparanta-Solin[4,5], Olof Solin[6,7,8], Sarita Forsback[6,7], Tove J. Grönroos[4,5], Chunlei Han[9], Francisco R. López-Picón[4,5, *], Hechun Xia[1, *]

1. Department of Neurosurgery, General Hospital of Ningxia Medical University, Yinchuan, Ningxia, China
2. Department of Neurosurgery, Affiliated Hospital of Hebei University, Baoding, Hebei, China
3. Department of Nuclear Medicine, General Hospital of Ningxia Medical University, Yinchuan, Ningxia, China
4. Preclinical Imaging Laboratory, Turku PET Centre, University of Turku, Turku, Finland
5. MediCity Research Laboratory, University of Turku, Turku, Finland
6. Radiopharmaceutical Chemistry Laboratory, Turku PET Centre, University of Turku, Turku, Finland
7. Department of Chemistry, University of Turku, Turku, Finland
8. Accelerator Laboratory, Turku PET Centre, Åbo Akademi University, Turku, Finland
9. Turku PET Centre, University of Turku, Turku, Finland

Xiaoqing Zhuang
Email: zhuangxq@nyfy.com.cn
Francisco R. López-Picón
Email: francisco.lopez@utu.f
Hechun Xia
Email: xhechun@nyfy.com.cn

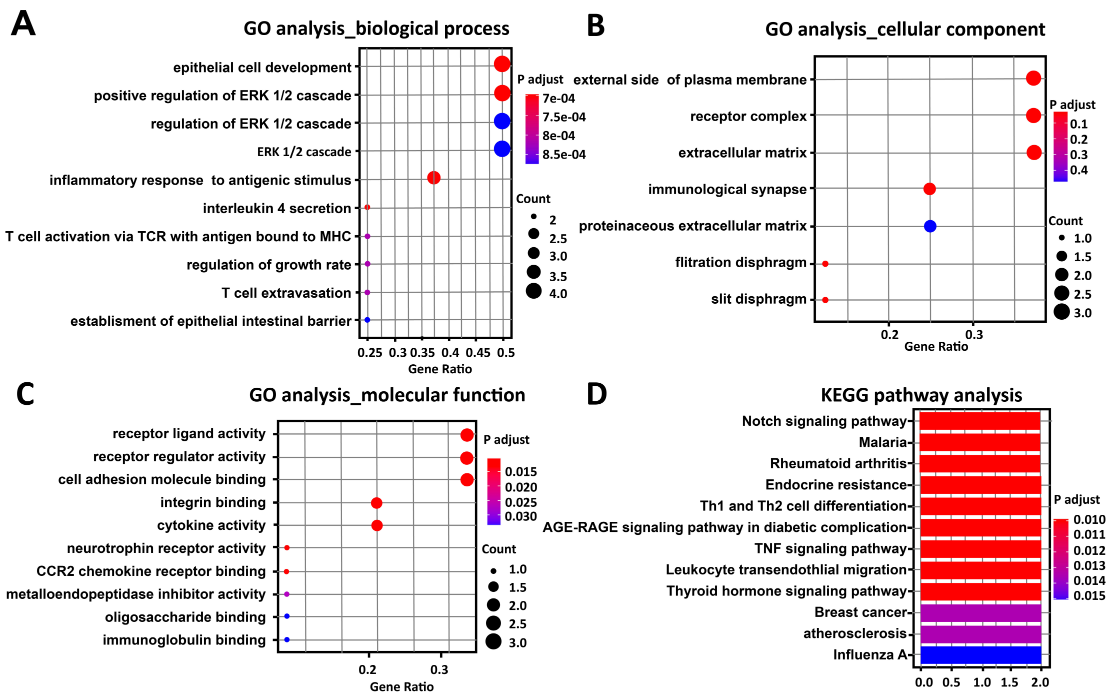

Fig. S1. GO enrichment and KEGG pathway analysis of differentially expressed proteins between the vehicle group and control group. a-c. The top 10 GO terms associated with biological process (**A**) cellular component (**B**) and molecular function (**C**) of differently expressed proteins are shown. D, KEGG pathway analysis for differently expressed proteins.

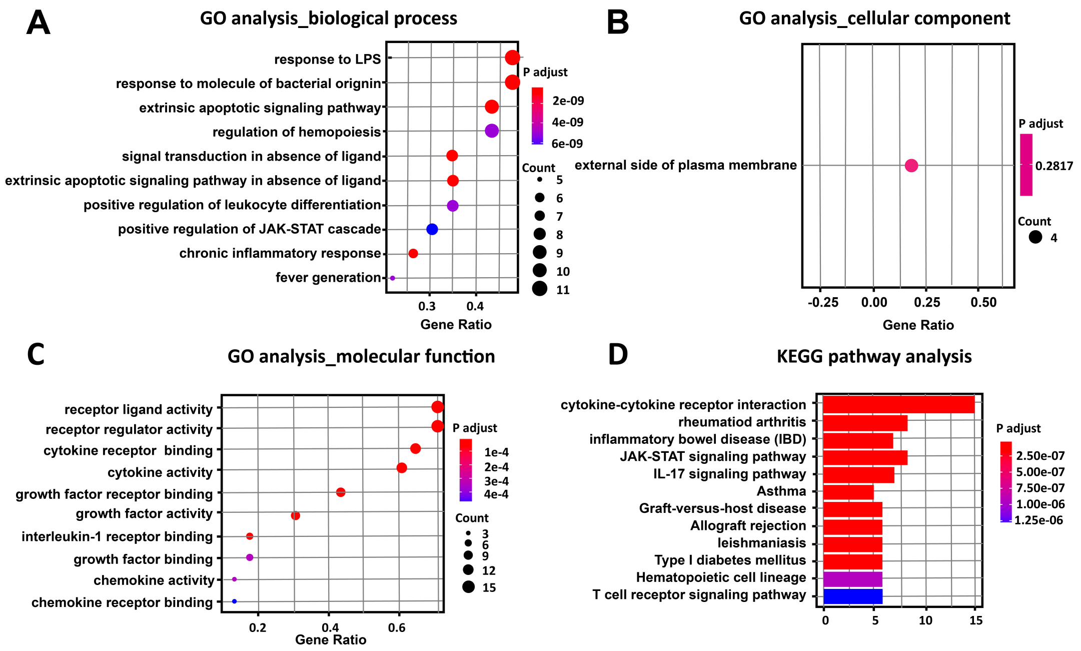

Fig. S2. GO enrichment and KEGG pathway analysis of differentially expressed proteins between the vehicle group and OEC group. (**A-C**) The top 10 GO terms associated with biological process (**A**), cellular component (**B**), and molecular function (**C**) of differently expressed proteins are shown. (**D**) KEGG pathway analysis for differently expressed proteins. LPS, Lipopolysaccharide.

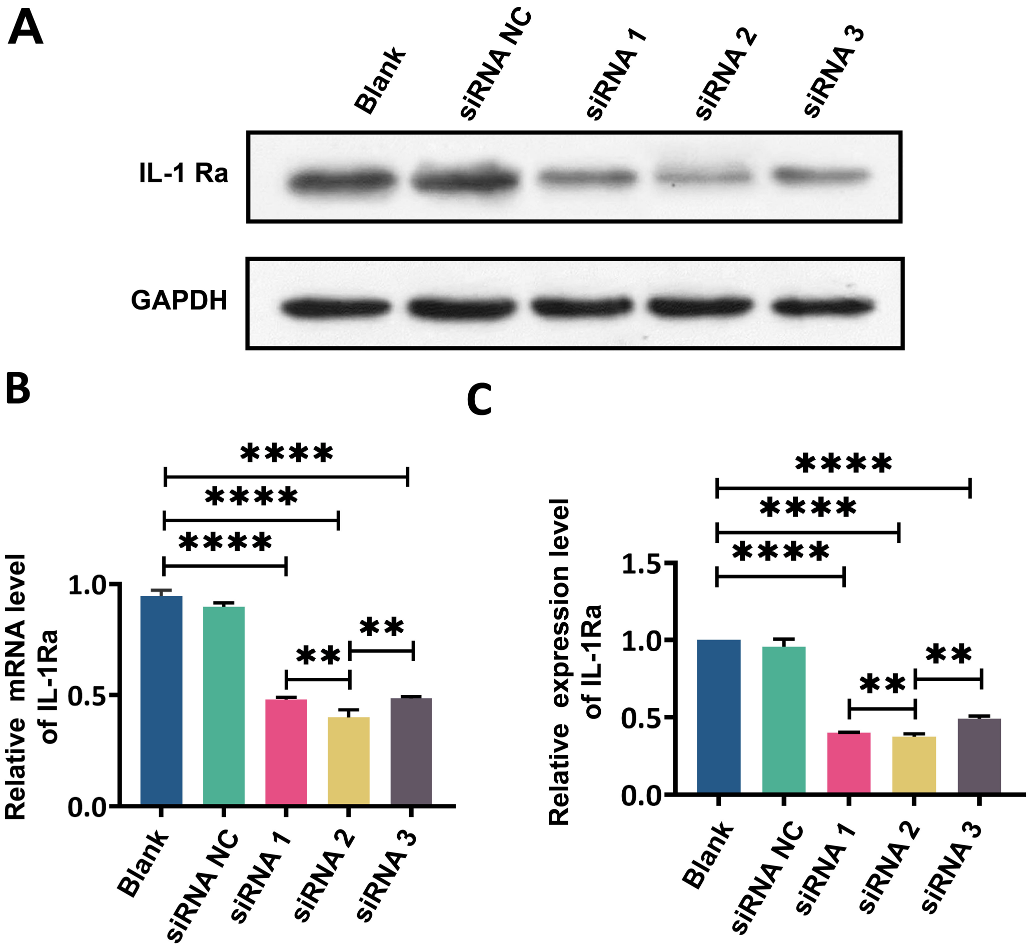

Fig. S3. Knockdown of IL-1Ra expression in OECs. (**A**) Western blot and quantification of IL-1 Ra expression in OECs following siRNA knockdown treatment. (**B**) Quantification of the mRNA level of IL-1Ra by PCR analysis. (**C**) Quantification of the IL-1Ra expression by western blot. **$p < 0.01$; ****$p < 0.0001$, n = 3.

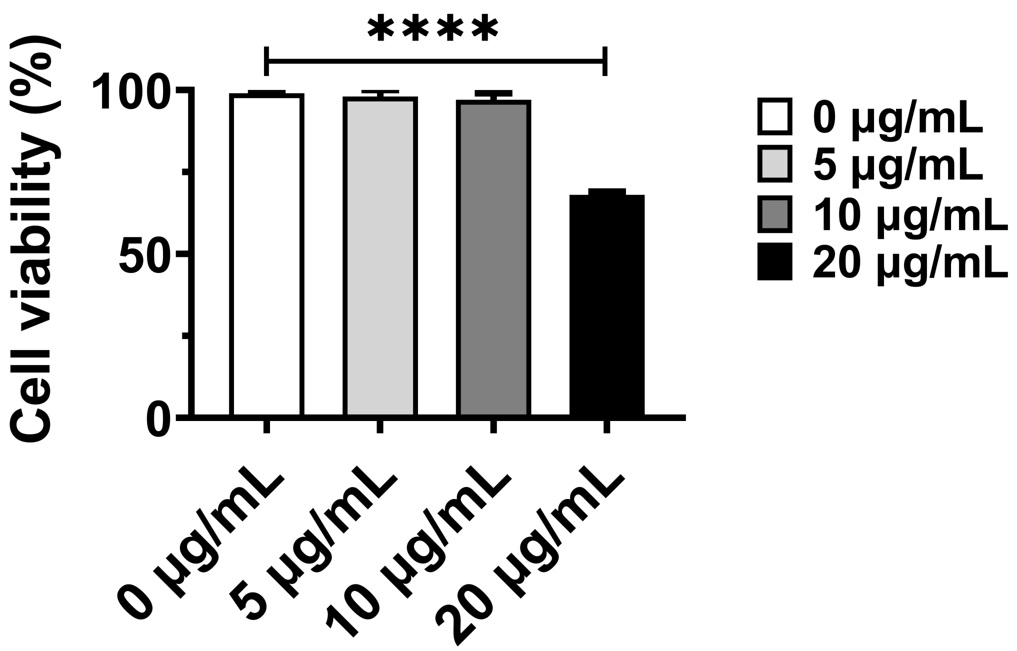

Fig. S4. Cell viability assay. The cytotoxicity of LPS to OECs was analysis with MTT assay. OECs were treated with different concentration of LPS. The data showed that 20 μg/mL could cause significant cytotoxicity to OECs. ***p < 0.001, n = 3.

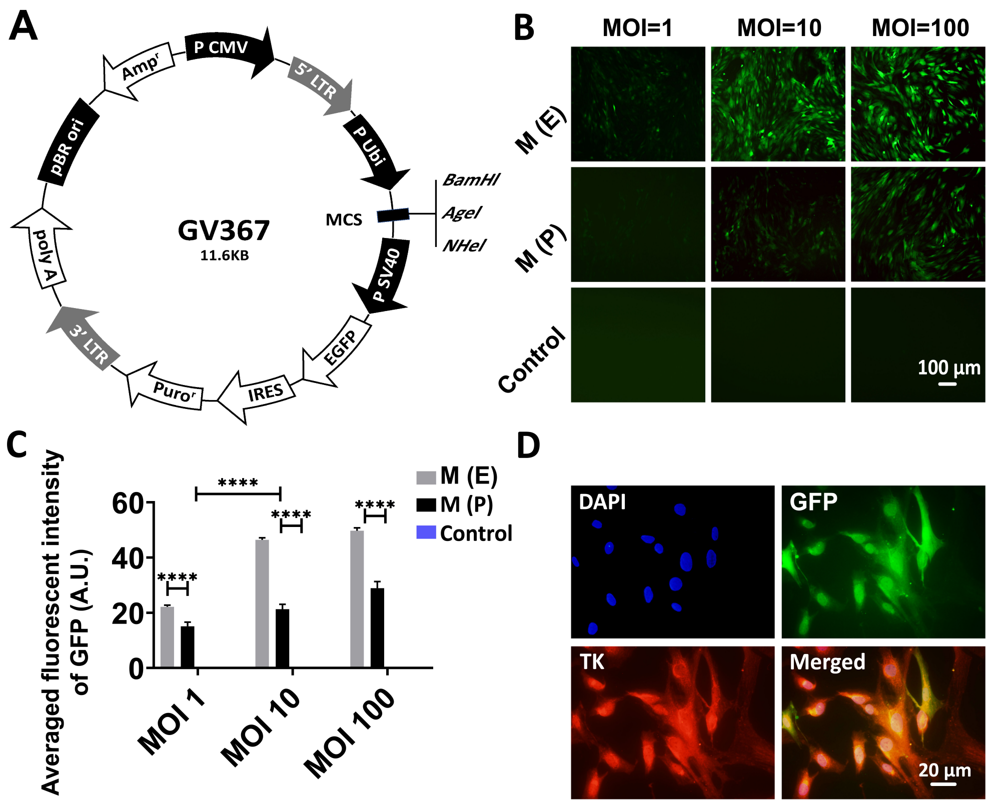

Fig. S5. Construction and identification of lentivirus-mediated HSV1-tk overexpression in OECs. (**A**) Schematic representation of lentivirus vector GV367. (**B**) Optimization of cell transfection conditions with LV-HSV1-TK. M (E): in complete medium supplement with Enhanced Infection Solution; M (P) in complete medium supplement with polybrene (scale = 100 μm). (**C**) Quantification of GFP fluorescence intensity under different transduction conditions. (**D**) LV-HSV1-TK (MOI = 10) facilitated HSV1-tk over-expression in OECs at 96 h post-transduction as verified by immunocytofluorescence. ****$p < 0.0001$, $n = 5$. Scale = 20 μm.

Table S1. The list of differentially expressed proteins between vehicle group and control group.

entrez ID	protein ID	Values_ vehicle group	Values_ control group	log2FC	p value
25464	ICAM-1	8281.25 ±1685.02	4973.31±689.14	0.735526	0.0135
24770	MCP-1	8097.86 ±1993.63	4686.61±592.64	0.788866	0.0240
116510	TIMP-1	14674.5943±186.82	1214.88±186.82	3.59334	0.0163
83781	Galectin-3	26711.18 ±6766.36	7379.32±2714.61	1.855743	9.58E-06
25454	GFR α-1	2295.74 ±245.82	1840.35±123.83	0.318823	0.0166
116479	JAM-A	907.79 ±151.03	579.43±134.28	0.646827	0.0119
25496	Notch-1	2475.36 ±313.46 1	1557.32±202.258	0.668238	0.0018
29492	Notch-2	5579.96 ±624.22	3547.58±469.951	0.65327	0.0010

n=5 per group

Table S2. The list of differentially expressed proteins between vehicle group and OEC group.

entrez ID	protein ID	Values_ OEC group	Values_ vehicle group	log2FC	p value
56822	B7-2	2199.48±318.69	2906.88±130.51	-0.40215	0.0081
310738	b-NGF	1263.14±292.04	1766.82±139.50	-0.4838	0.0220
116630	GM-CSF	2127.27±88.23	2713.69±129.25	-0.3511	0.0001
25712	IFNg	2025.79±96.39	2542.39±120.14	-0.32755	0.0001
24493	IL-1α	1125.08±60.51	1423.16±95.01	-0.3388	0.0012
24494	IL-1β	1662.91±235.28	2094.22±51.97	-0.33252	0.0198
116562	IL-2	1148.19±146.36	1445.81±128.67	-0.33225	0.0160
287287	IL-4	2572.32±62.57	3420.95±228.74	-0.41119	0.0011
25325	IL-10	1649.18±142.12	2017.38±142.60	-0.29057	0.0064
60665	LIX	2922.85±113.71	3649.18±80.91	-0.3201	1.32E-05
25266	PDGF-AA	3894.12±360.12	4953.65±358.91	-0.34711	0.0031
24684	Prolactin R	843.73±149.78	1210.77±91.33	-0.52054	0.0046
24835	TNF α	2283.99±116.40	2907.36±212.95	-0.34802	0.0019
83785	VEGF	778.06±144.79	1009.01±69.72	-0.37456	0.0296
362505	CTACK	1386.02±101.22	1728.56±163.10	-0.31842	0.0098
29397	Eotaxin	1557.26±128.15	1929.14±166.87	-0.30877	0.0085
64535	FGF-BP	926.86±85.90	1168.37±148.41	-0.33375	0.0284
60582	IL-1 ra	666.09±235.56	175.97±88.98	1.914345	0.0109
24495	IL-3	963.315±32.83	1158.62±104.75	-0.26609	0.0175
116479	JAM-A	532.45±166.11	907.79±151.03	-0.76861	0.0103
81527	Neuropilin-2	849.05 ±56.51	1071.98±134.67	-0.33599	0.0258
25496	Notch-1	1607.81±380.82	2475.36±313.46	-0.62224	0.0083
29492	Notch-2	3682.22±605.67	5579.96±624.22	-0.59955	0.0024

n=5 per group

Table S3. Gene ontology (GO) functional enrichment analysis for the differentially expressed proteins (DEPs) between vehicle group and OEC group.

ID	Description	P value	p. adjust	Gene ID	Enrich factor
**Biological process**					
GO:0032496	response to LPS (lipopolysaccharide)	2.39E-12	1.12E-09	Cd86, Ngf, Csf2, Ifng, Il1a, Il1b, Il10, Cxcl6, Tnf, Il1rn, Notch1	18.96427
GO:0071219	cellular response to molecule of bacterial origin	1.39E-08	6.03E-07	Cd86, Csf2, Ifng, Il1b, Il10, Cxcl6, Tnf	22.86333
GO:0097191	extrinsic apoptotic signaling pathway	9.21E-14	7.21E-11	Ngf, Csf2, Ifng, Il1a, Il1b, Il2, Il4, Tnf, Vegfa, Il3	34.10075
GO:1903708	positive regulation of hemopoiesis	8.29E-11	1.22E-08	Csf2, Ifng, Il1b, Il2, Il4, Tnf, Il3, Notch2	31.435
GO:0038034	signal transduction in absence of ligand	4.36E-14	5.12E-11	Ngf, Csf2, Il1a, Il1b, Il2, Il4, Tnf, Il3	79.39353
GO:0097192	extrinsic apoptotic signaling pathway in absence of ligand	4.36E-14	5.12E-11	Ngf, Csf2, Il1a, Il1b, Il2, Il4, Tnf, Il3	79.39353
GO:1902107	positive regulation of leukocyte differentiation	1.34E-11	3.49E-09	Csf2, Ifng, Il1b, Il2, Il4, Tnf, Il3, Notch2	39.44392
GO:0002544	chronic inflammatory response	1.06E-12	6.25E-10	Il1b, Il4, Il10, Tnf, Ccl11, Il1rn	160.1559
GO:0001660	fever generation	9.77E-12	3.28E-09	Il1a, Il1b, Tnf, Il1rn, Il3	241.9022
**Molecular function**					

GO:0048018	receptor ligand activity	5.36E-21	1.36E-19	Ngf, Csf2, Ifng, Il1a, Il1b, Il2, Il4, Il10, Cxcl6, Pdgfa, Tnf, Vegfa, Ccl27, Ccl11, Il1rn, Il3	27.18185
GO:0030545	receptor regulator activity	1.69E-20	3.21E-19	Ngf, Csf2, Ifng, Il1a, Il1b, Il2, Il4, Il10, Cxcl6, Pdgfa, Tnf, Vegfa, Ccl27, Ccl11, Il1rn, Il3	25.31127
GO:0005126	cytokine receptor binding	9.78E-22	3.72E-20	Ngf, Csf2, Ifng, Il1a, Il1b, Il2, Il4, Il10, Cxcl6, Tnf, Vegfa, Ccl27, Ccl11, Il1rn, Il3	38.04873
GO:0005125	cytokine activity	8.40E-23	6.39E-21	Csf2, Ifng, Il1a, Il1b, Il2, Il4, Il10, Cxcl6, Tnf, Vegfa, Ccl27, Ccl11, Il1rn, Il3	58.18374
GO:0070851	growth factor receptor binding	1.31E-15	1.99E-14	Csf2, Il1a, Il1b, Il2, Il4, Il10, Pdgfa, Vegfa, Il1rn/Il3	51.80343
GO:0008083	growth factor activity	5.54E-10	7.02E-09	Ngf, Csf2, Il2, Il4, Pdgfa, Vegfa, Il3	36.2624
GO:0005149	interleukin-1 receptor binding	7.84E-09	8.51E-08	Il1a, Il1b, Il1rn, Il3	163.4686
GO:0019838	growth factor binding	4.15E-05	0.00035	Pdgfa, Vegfa, Fgfbp1, Il1rn	20.15366
GO:0042379	chemokine receptor binding	6.12E-05	0.000465	Cxcl6, Ccl27, Ccl11	38.71625
GO:0008009	chemokine activity	1.65E-05	0.000157	Cxcl6, Ccl27, Ccl11	59.64395
**Cellular component**					
GO:0009897	external side of plasma membrane	0.000655	0.028174	Cd86, Ifng, Il4, Tnf	9.820785

Notes: Category refers to the GO functional categories.

Table S4. The map of protein array.

CYT-3

	1	2	3	4	1	2	3	4	1	2	3	4
**A**	POS1				POS2				NEG			
**B**	CD86				Beta-NGF				CXCL1			
**C**	CINC-2				CINC-3				CNTF			
**D**	Fractalkine				GM-CSF				ICAM-1			
**E**	IFN-γ				IL-1 α				IL-1β			
**F**	IL-2				IL-4				IL-6			
**G**	IL-10				IL-13				LIX			
**H**	L-Selectin				MCP-1				PDGF-AA			
**I**	Prolactin R				RAGE				CXCL-7			
**J**	TIMP-1				TNF-α				VEGF-A			

CYT-4

	1	2	3	4	1	2	3	4	1	2	3	4
**A**	POS1				POS2				CD137			
**B**	Activin A				Adiponectin				B7-1			
**C**	CD48				CCL27				Decorin			
**D**	Eotaxin-1				EphA5				Erythropoietin			
**E**	FGF-BP				Flt-3 Ligand				Galectin-1			
**F**	Galectin-3				Gas-1				GFR α-1			
**G**	Gp130				HGF				IL-1R6			
**H**	IL-1Ra				IL-2R α				IL-3			
**I**	IL-7				IL-17F				IL-22			
**J**	JAM-A				MIP-1α				Neuropilin-1			
**K**	Neurolilin-2				Nope				Notch-1			
**L**	Notch-2				P-Cadherin				Prolactin			
**M**	RANTES				SCF				TIM-1			
**N**	TIMP-2				TREM-1				TWEAKR			

Table S5. Pathway enrichment analysis for the differentially expressed proteins (DEPs) between SCI group and control group.

Category	Term	p value	p.adjust	geneID	Enrich_factor
rno04330	Notch signaling pathway	0.0005346	0.009995	Notch1, Notch2	54.87179
rno05144	Malaria	0.0007118	0.009995	Icam1, Ccl2	47.55556
rno05323	Rheumatoid arthritis	0.0016665	0.009995	Icam1, Ccl2	31.01449
rno01522	Endocrine resistance	0.0017026	0.009995	Notch1, Notch2	30.681
rno04658	Th1 and Th2 cell differentiation	0.0017391	0.009995	Notch1, Notch2	30.35461
rno04933	AGE-RAGE signaling pathway in diabetic complications	0.0020443	0.009995	Icam1, Ccl2	27.97386
rno04668	TNF signaling pathway	0.0024161	0.009995	Icam1, Ccl2	25.70571
rno04670	Leukocyte transendothelial migration	0.002591	0.009995	Icam1, F11r	24.81159
rno04919	Thyroid hormone signaling pathway	0.002726	0.009995	Notch1, Notch2	24.18079
rno05224	Breast cancer	0.004312	0.013451	Notch1, Notch2	19.14989
rno05164	Influenza A	0.0056451	0.015159	Icam1, Ccl2	16.68616
rno04514	Cell adhesion molecules (CAMs)	0.0059717	0.015159	Icam1, F11r	16.21212
rno05206	MicroRNAs in cancer	0.0137684	0.032454	Notch1, Notch2	10.52891

Notes: Category refers to the pathway functional categories.

Table S6. Pathway enrichment analysis for the differentially expressed proteins (DEPs) between vehicle group and OEC group.

Category	Term	p value	p. adjust	Gene ID	Enrich factor
rno04060	Cytokine-cytokine receptor interaction	8.97E-19	9.42E-17	Ngf, Csf2, Ifng, Il1a, Il1b, Il2, Il4, Il10, Cxcl6, Prlr, Tnf, Ccl27, Ccl11, Il1rn, Il3	22.72969
rno05323	Rheumatoid arthritis	2.37E-11	1.25E-09	Cd86, Csf2, Ifng, Il1a, Il1b, Cxcl6, Tnf, Vegfa	35.44513
rno05321	Inflammatory bowel disease (IBD)	1.25E-10	4.37E-09	Ifng, Il1a, Il1b, Il2, Il4, Il10, Tnf	43.23232
rno04630	JAK-STAT signaling pathway	1.61E-09	3.57E-08	Csf2, Ifng, Il2, Il4, Il10, Pdgfa, Prlr/Il3	21.0384
rno04657	IL-17 signaling pathway	1.70E-09	3.57E-08	Csf2, Ifng, Il1b, Il4, Cxcl6, Tnf, Ccl11	30.03509
rno05310	Asthma	1.21E-08	1.94E-07	Il4, Il10, Tnf, Ccl11, Il3	61.76046
rno05332	Graft-versus-host disease	1.29E-08	1.94E-07	Cd86, Ifng, Il1a, Il1b, Il2, Tnf	34.44668
rno05330	Allograft rejection	1.66E-08	2.11E-07	Cd86, Ifng, Il2, Il4, Il10, Tnf	33.05019
rno05140	Leishmaniasis	1.81E-08	2.11E-07	Ifng, Il1a, Il1b, Il4, Il10, Tnf	32.60952
rno04940	Type I diabetes mellitus	2.67E-08	2.81E-07	Cd86, Ifng, Il1a, Il1b, Il2, Tnf	30.57143
rno04640	Hematopoietic cell lineage	8.57E-08	8.18E-07	Csf2, Il1a, Il1b, Il4, Tnf, Il3	25.21355
rno04660	T cell receptor signaling pathway	1.46E-07	1.28E-06	Csf2, Ifng, Il2, Il4, Il10, Tnf	23.07278

Notes: Category refers to the pathway functional categories.

Table S7. Gene ontology (GO) functional enrichment analysis for the differentially expressed proteins (DEPs) between vehicle group and control group.

ID	Description	p value	p. adjust	Gene ID	Enrich factor
**Biological process**					
GO:0002064	epithelial cell development	2.20E-06	0.000699	Icam1, F11r, Notch1, Notch2	36.93776
GO:0070374	positive regulation of ERK1 and ERK2 cascade	2.42E-06	0.000699	Icam1, Ccl2, Notch1, Notch2	36.04049
GO:0070372	regulation of ERK1 and ERK2 cascade	7.03E-06	0.000872	Icam1, Ccl2, Notch1, Notch2	27.56037
GO:0070371	ERK1 and ERK2 cascade	8.81E-06	0.000875	Icam1, Ccl2, Notch1, Notch2	26.02924
GO:0002437	inflammatory response to antigenic stimulus	2.81E-06	0.000699	Icam1, Notch1, Notch2	99.64925
GO:0072602	interleukin-4 secretion	2.65E-06	0.000699	Notch1, Notch2	741.8333
GO:0002291	T cell activation via T cell receptor contact with antigen bound to MHC molecule on antigen presenting cell	3.71E-06	0.000736	Icam1, Lgals3	635.8571
GO:0040009	regulation of growth rate	4.94E-06	0.000818	Notch1, Notch2	556.375
GO:0072683	T cell extravasation	6.35E-06	0.000872	Icam1, Ccl2	494.5556
GO:0090557	establishment of endothelial intestinal barrier	7.94E-06	0.000875	Icam1, F11r	445.1
**Cellular component**					
GO:0036056	filtration diaphragm	0.0034508	0.024155	F11r	289.4063
GO:0036057	slit diaphragm	0.0034508	0.024155	F11r	289.4063

GO:0001772	immunological synapse	9.09E-05	0.00382	Icam1, Lgals3	136.1912
GO:0009897	external side of plasma membrane	0.0002885	0.005938	Icam1, Lgals3, Gfra1	21.17607
GO:0043235	receptor complex	0.0004241	0.005938	Gfra1, Notch1, Notch2	18.57152
GO:0031012	extracellular matrix	0.0006598	0.006928	Icam1, Timp1, Lgals3	15.96724
GO:0005578	proteinaceous extracellular matrix	0.0077777	0.046666	Timp1, Lgals3	14.47031
**Molecular function**					
GO:0048018	receptor ligand activity	0.000847	0.011758	Ccl2, Timp1, Lgals3	14.65271
GO:0030545	receptor regulator activity	0.001042	0.011758	Ccl2, Timp1, Lgals3	13.64435
GO:0050839	cell adhesion molecule binding	0.0011703	0.011758	Icam1, Gfra1, F11r	13.10873
GO:0005178	integrin binding	0.0012059	0.011758	Icam1, Gfra1	37.43142
GO:0005125	cytokine activity	0.0029236	0.016289	Ccl2, Timp1	23.89689
GO:0005030	neurotrophin receptor activity	0.0023623	0.015355	Gfra1	422.975
GO:0048020	CCR chemokine receptor binding	0.0182965	0.039642	Ccl2	54.22756
GO:0008191	metalloendopeptidase inhibitor activity	0.0051905	0.025304	Timp1	192.2614
GO:0070492	oligosaccharide binding	0.0080117	0.033397	Lgals3	124.4044
GO:0019865	immunoglobulin binding	0.0089506	0.033397	Lgals3	111.3092

Notes: Category refers to the GO functional categories.

Table S8. List of siRNA sequences used in this article.

Name	Sense (5'-3') primer
si RNA-1	GCUCAUUGCUGGGUACUUATT
si RNA-2	AUCAGUGAUGUUAACCUCCTC
si RNA-3	UUGUGCAGAGGAACCAUCCTG

Table S9. List of qPCR sequences used in this article.

**Gene symbol**	**Primers sequence**
Rattus GAPDH-F	*GATGACATCAAGAAGGTGGTGA*
Rattus GAPDH-R	*ACCCTGTTGCTGTAGCCATATTC*
Rattus IL-1 RA-F	*GACATGGTGCCTATTGACTTTC*
Rattus IL-1 RA-R	*GATTCGAAGCTGGTGGTAGG*
